# Femoral neck fracture: the reliability of radiologic classifications

**DOI:** 10.1186/s12891-022-05007-3

**Published:** 2022-01-25

**Authors:** Gianpiero Cazzato, Maria Serena Oliva, Giulia Masci, Raffaele Vitiello, Alessandro Smimmo, Maria Rosaria Matrangolo, Osvaldo Palmacci, Stefano D’Adamio, Antonio Ziranu

**Affiliations:** 1 RomaPRO Center for Hip and Knee Arthroplasty, Polo Sanitario San Feliciano, Rome, Italy; 2 Department of Orthopaedics and Traumatology, Fondazione Policlinico Universitario A. Gemelli IRCCS - Università Cattolica del Sacro Cuore, Rome, Italy; 3 UOC Orthopaedic and Traumatology, Children’s Hospital Bambino Gesù, Rome, Italy

**Keywords:** Hip fractures, Femoral neck fracture, Femoral fractures’ classification, Reliability

## Abstract

**Background:**

Femoral neck fractures (FNF) are one of the most common injury in the elderly. A valid radiographic classification system is mandatory to perform the correct treatment and to allow surgeons to facilitate communication. This study aims to evaluate reliability of 2018 AO/OTA Classification, AO/OTA simplified and Garden classification.

**Methods:**

Six Orthopaedic surgeons, divided in three groups based on trauma experience, evaluated 150 blinded antero-posterior and latero-lateral radiography of FNF using Garden classification, 2018 AO/OTA and simplified AO/OTA classification. One month later, the radiographs were renumbered and then each observer performed a second evaluation of the radiographs. The Kappa statistical analysis was used to determine the reliability of the classifications. Cohen’s Kappa was calculated to determine intra and inter observer reliability. Fleiss’ Kappa was used to determine multi-rater agreement.

**Results:**

The k values of interobserver reliability for Garden classification was from 0,28 to 0,73 with an average of 0,49. AO classification showed reliability from 0,2 to 0,42, with average of 0,30. Simplified AO/OTA classification showed a reliability from 0,38 to 0,58 with an average of 0,48.

The values of intra observer reliability for Garden classification was from 0,48 to 0,79 with an average of 0,63. AO classification showed reliability from 0,2 to 0,64 with an average of 0,5. Simplified AO/OTA classification showed a reliability from 0,4 to 0,75 with an average of 0,61.

**Conclusion:**

The revised 2018 AO/OTA classification simplified the previous classification of intracapsular fracture but remain unreliable with only fair interobserver reliability. The simplified AO/OTA classification show a reliability similar to Garden classification, with a moderate interobserver reliability. The experience of the surgeons seems not to improve reliability. No classification has been shown to be superior in terms of reliability.

## Background

Proximal femur fracture is one of the most common type of fracture in the elderly. It occurs in 18% of women and in 6% of men worldwide [1]. It is caused by accidental falls in elderly patients, due to osteoporosis [2]. The incidence of proximal femur fracture has raised worldwide in the last two decades along with the increase in the average age of the population. In fact, the global number of hip fractures is expected to increase from 1.26 million in 1990 to 4.5 million by the year 2050 [1].

The incidence of femoral neck fractures (FNF) is approximately equal to the incidence of pertrochanteric fractures, in combination making up over 90% of all proximal femur fractures [3].

In Italy, hip fractures occurred in people over 65 years increased from 89,601 to 94,525 during the period from 2007 to 2014 [4]. This leads to an increasing number of hospital admission and hospitalization costs [5]. Furthermore hip fractures affect the quality of life of patients [6]. For this reason it is important to reach a fast and correct diagnosis and perform an adequate and prompt treatment to reduce post-operative complications [7] and mortality [8].

The treatment of choice, in almost all of the cases, is surgical. The choice of a specific treatment option is based on the stability and orientation of the fracture and patient factors such as age, function, and bone quality [9, 10]. For unstable FNF the treatment of choice is hip replacement (total hip arthroplasty or hemiarthroplasty) instead for stable FNF, the most used treatment is the internal fixation with cannulated screws or with other hip implants [11].

Radiographic FNF classification helps with clinical decision making, communication, and research on prognosis and treatment [12]. The most common classification used for intracapsular FNF are the Garden Classification and the AO/OTA classification. These classification systems are based on 2-dimensional X-ray images. Garden classified femoral neck fractures into four types based on displacement on the anteroposterior radiograph [13, 14]. A type I fracture is an incomplete or valgus-impacted fracture. A type II fracture is a complete fracture without displacement of the fracture fragments. A type III fracture is a complete fracture with partial displacement of fracture fragments. A type IV fracture is a complete fracture with total displacement of the fracture fragments, allowing the femoral head to rotate back to an anatomic position [9]. The AO/OTA classification system is organized into hierarchies of severity as the descriptions generally proceed from simple to multifragmentary fractures [15]. Fractures of the femoral head have been classified as subcapital with minimal or no displacement (Type B1), transcervical (Type B2), or displaced subcapital fractures (Type B3). Each of these types has a subclassification [16]. In clinical practice AO/OTA classification is usually simplified considering only the three categories (B1, B2, B3).

The aim of the study is to assess the reliability of these classifications by examining intra- and interobserver agreement of trauma surgeons and how the reliability depends on observers’ experience.

## Methods

In this retrospective study were included patients admitted to a single institution from January 2017 to December 2019 for FNF.

The inclusion criteria was femoral neck fracture in a patient aged 18 years or more.

The exclusion criteria were: incomplete series of preoperative radiography (it was requested digital files of antero-posterior projection of the pelvis and hip in lateral projection), advanced hip osteoarthritis, previous contralateral side femoral neck fracture or contralateral prosthetic replacement, hip dysplasia, associated pelvic fractures. Pathologic fractures were excluded too.

The final sample size consisted of 150 patients, including 57 men and 93 women, with an average age of 75,6 years. The hip involved in 43% of cases was the right. Of this sample, 49 patients underwent CRIF or ORIF surgery, 101 patients underwent prosthesis surgery (in 4 cases, a computed tomography was used for the surgical choice).

All possible patient identification marks were obscured on the radiographs. The radiographs were subsequently numbered and were analyzed, by 6 observers: 2 experienced trauma surgeons (GC and SD), 2 junior trauma surgeons (GM and MSO) and 2 orthopaedic trauma residents (AS and MM). All observers were familiar with the classifications analyzed, and all of them were equipped with the classifications’ definitions and schemes. Surgeons with different experience were chosen to assess how much experience could affect reproducibility. Radiographs were classified according to 2018 edition AO/OTA classification, 2018 edition AO/OTA simplified (only B1, B2, B3) and Garden classification.

**Fig. 1:**
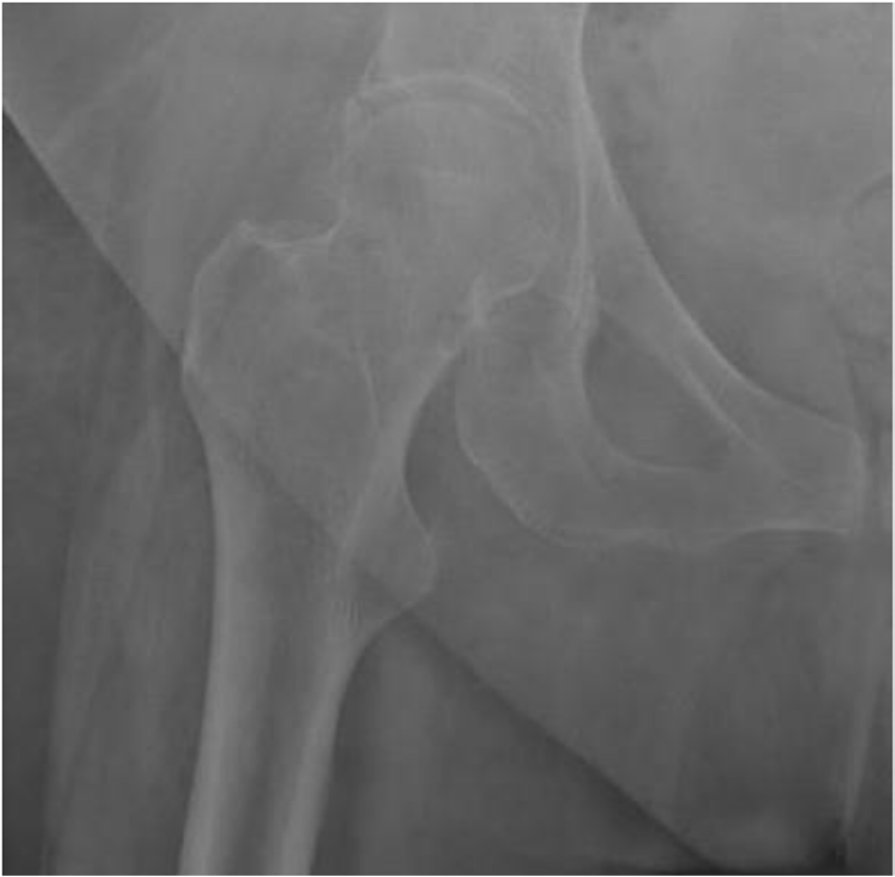
Example of blinded radiographs included in the study. (1) Femural neck fracture with high intra and inter observer reliability. (2) Femural neck fracture with low intra and inter observer reliability

**Fig. 2:**
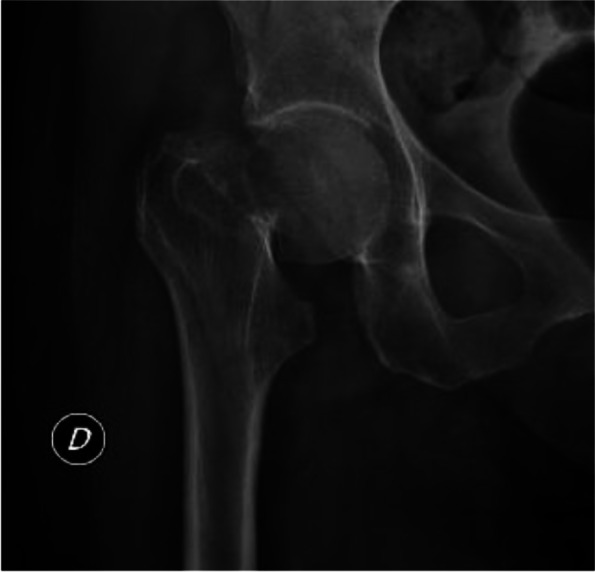
Example of blinded radiographs included in the study. (1) Femural neck fracture with high intra and inter observer reliability. (2) Femural neck fracture with low intra and inter observer reliability

Each observer was required to make a first classification of the radiographs in AP and LL projection, noting the results in a specific grid. At the end of the observation, the grid was archived without sharing it with the other observers. One month later, the radiographs were renumbered and then each observer performed a second evaluation of the radiographs (Figs. 1 and 2).

The Kappa statistical analysis was used to determine the reliability of the classifications. Cohen’s Kappa was calculated to determine intra and inter observer reliability. Fleiss’ Kappa was used to calculate the multi rater reliability of more and less experienced trauma surgeons.

We used the interpretation of intra and interobserver variability using the Landis and Koch criteria: a k values of 0.00–0.20 considered slight agreement; 0.21–0.40, fair agreement; 0.41–0.60 moderate agreement; 0.61–0.80, substantial agreement; 0.81–1.00, almost perfect agreement [14].

## Results

### Interobserver reliability

Cohen kappa’s values of interobserver reliability of AO/OTA, AO/OTA simplified, and Garden classification based on X ray are noted in Table 1 – 2 – 3. The values of interobserver reliability for Garden classification was from 0,28 [0,17–0,39 CI] to 0,73 [0,65–0,82 CI] with an average of 0,49. AO classification showed reliability from 0,2 [0,11–0,29 CI] to 0,42 [0,33–0,52 CI], with average of 0,30. Simplified AO/OTA classification showed a reliability from 0,38 [0,26–0,50 CI] to 0,58 [0,47-0,69] with an average of 0,48.

**Table 1:** Inter-observer reliability of Garden Classification. K Value [95% CI] - %Agreement

	GC	SD	GM	MSO	AS	MM
GC	///	0.57 [0.47–0.67] – 68%	0.43 [0.33–0.54] – 57%	0.49 [0.39–0.60] – 62%	0.58 [0.48–0.68] – 68%	0.73 [0.65–0.82] – 80%
SD		///	0.37 [0.26–0.48] – 53%	0.48 [0.38–0.59] – 61%	0.54 [0.44–0.64] – 65%	0.64 [0.54–0.73] – 73%
GM			///	0.54 [0.54–0.64] – 65%	0.28 [0.17–0.39] – 46%	0.35 [0.24–0.46] – 51%
MSO				///	0.4 [0.29–0.5] – 55%	0.45 [0.34–0.55] – 59%
AS					///	0.54 [0.44–0.64] – 65%
MM						///

**Table 2:** Inter-observer reliability of AO/OTA Classification. K Value [95% CI] - %Agreement

	GC	SD	GM	MSO	AS	MM
GC	///	0.42 [0.33–0.52] -51%	0.37 [0.28–0.46] – 46%	0.32 [0.23–0.42] – 42%	0.25 [0.16–0.34] – 36%	0.38 [0.28–0.47] – 47%
SD		///	0.39 [0.29–0.48] – 47%	0.27 [0.18–0.36] – 37%	0.2 [0.11–0.29] – 31%	0.29 [0.2–0.38] – 39%
GM			///	0.37 [0.28–0.46] – 46%	0.24 [0.15–0.33] – 35%	0.26 [0.17–0.35] – 37%
MSO				///	0.21 [0.12–0.29] – 32%	0.35 [0.25–0.44] – 44%
AS					///	0.22 [0.13–0.31] – 33%
MM						///

**Table 3:** Inter-observer reliability of Simplified AO/OTA Classification. K Value [95% CI] - %Agreement

	GC	SD	GM	MSO	AS	MM
GC	///	0.58 [0.47–0.69] – 72%	0.45 [0.33–0.57] – 63%	0.56 [0.45–0.67] – 71%	0.50 [0.39–0.61] – 67%	0.5 [0.39–0.61] – 67%
SD		///	0.49 [0.38–0.6] – 66%	0.54 [0.43–0.65] – 69%	0.44 [0.32–0.56] – 63%	0.44 [0.32–0.56] – 63%
GM			///	0.46 [0.34–0.58] – 64%	0.45 [0.33–0.57] – 63%	0.38 [0.26–0.50] -59%
MSO				///	0.48 [0.37–0.59] – 65%	0.49 [0.38–0.60] – 66%
AS					///	0.48 [0.37–0.59] – 65%
MM						///

We also analyzed the agreement between observers, dividing them in groups according to their trauma experience: trauma surgeon, young trauma surgeon and resident. There were no significant differences in agreement between observer groups (Table 4). The results shows a moderate agreement as regards both Garden classification and simplified AO/OTA classification; the mean K value was lower when considering AO/OTA classification; results demonstrated a fair agreement.

**Table 4:** Groups’ Kappa values for inter observer agreement of Classifications. K Value [95% CI] - %Agreement

	Garden		AO/OTA		Simplified AO/OTA	
	Junior trauma surgeon	Resident	Junior trauma surgeon	Resident	Junior trauma surgeon	Resident
Trauma surgeon	0.48 [0.42–0.54] – 61%	0.60 [0.53–0.67] – 70%	0.36 [0.30–0.41] – 45%	0.29 [0.25–0.34] – 39%	0.51 [0.44–0.58] – 68%	0.49 [0.42–0.56] – 66%
Junior trauma surgeon		0.43 [0.36–0.49] – 57%		0.27 [0.22–0.32] – 37%		0.46 [0.38–0.53] – 64%

### Intra observer reliability

Cohen kappa’s values of intra observer reliability of AO/OTA, AO/OTA simplified, and Garden classification based on X ray are noted in Table 5. The values of intra observer reliability for Garden classification was from 0,48 [0,37–0,58 CI] to 0,79 [0,71–0,87 CI] with an average of 0,63. AO/OTA classification showed reliability from 0,2 [0,13–0,3 CI] to 0,64 [0,56–0,73 CI] with an average of 0,5. Simplified AO/OTA classification showed a reliability from 0,4 [0,28–0,52 CI] to 0,75 [0,66-0,84] with an average of 0,61.

**Table 5:** Intra-observer reliability of classifications. K Value [95% CI] - %Agreement

	Garden	AO/OTA	Simplified AO/OTA
GC	0.62 [0.52–0.71] – 71%	0.64 [0.56–0.73] – 69%	0.75 [0.66–0.84] – 83%
SD	0.59 [0.45–0.73] – 69%	0.52 [0.39–0.65] – 59%	0.62 [0.47–0.77] – 75%
GM	0.58 [0.48–0.68] – 69%	0.55 [0.46–0.64] – 61%	0.62 [0.52–0.72] – 75%
MSO	0.71 [0.62–0.80] – 78%	0.53 [0.43–0.62] – 50%	0.65 [0.55–0.75] – 77%
AS	0.48 [0.37–0.58] – 61%	0.2 [0.13–0.3] – 33%	0.4 [0.28–0.52] – 60%
MM	0.79 [0.71–0.87] – 84%	0.58 [0.49–0.67] – 64%	0.65 [0.55–0.75] – 77%

## Discussion

Successful treatment starts by an adequate classification of pathology and an accurate evaluation of the clinical condition of the patient (age, comorbidities) that guides surgeons in choosing the correct management and communication.

Ideally, a classification system should be easily applicable, highly reliable, comprehensive, highly reproducible; in many cases it indicates outcomes. Regarding proximal femur fractures there is still no agreement on a universally accepted, reliable classification, and this can stimulate debate regarding the appropriate treatment options. Any classification system used should aim to possess a high degree of inter-observer and intra-observer reliability facilitating the communication of patient’s conditions providing a clear guidance for the treatment of patients [17].

A valid classification allows surgeons to determine the correct treatment and predict outcomes. In fact, femoral neck fractures were firstly classified by Waldenström in 1924 in “stable” and “unstable”. In literature, reliability of this classification was widely analyzed; datas show that it’s higher than in the others, because it considers only two level, instead of four and seven level respectively in Garden and AO classification, reducing possible bias [18] [19].

In the past the Pauwels classification has also been studied, resulting in a poorly reliability classification and therefore no longer used in daily clinical practice [20] [21].

In this paper we have studied the inter-observer and intra-observer agreement evaluation of three different classification systems. Six orthopedic trauma surgeons, with different years of experience (two young trauma surgeons, two residents, two trauma surgeons) graded 150 radiographs of proximal femur fractures using Garden classification and 2018 AO classification, complete and simplified. We decided to not use the Waldenström and Pauwels classifications because these are not used a day in the clinical practice.

The inter-observer reliability obtained in Garden classification was moderate, as regards simplified AO/OTA was moderate too (average k value was 0,49 and 0,48 respectively). Inter-observer reliability lessens to fair with an average k value of 0.30 when considering AO classification.

In literature the interobserver agreement of Garden classification varies from fair to moderate [21–23]. Our results demonstrated for the Garden classification an higher reliability compared to the previous study: Masionis et al., Gaspar et al. and Van Embden et all found a k value respectively of 0,33, 0,41 and 0,31 [18, 21, 22].

We found a substantial intra-observer reliability in Garden and simplified AO/OTA classification, (mean k value was 0,63 and 0,61 respectively). Intra-observer reliability lessens to moderate with a mean k value of 0,50 when considering AO/OTA classification.

Even for Garden’s intraobserver reliability, we found an higher k value compared with previous studies: Masionis et al. found an intraobserver reliability from 0,40 to 0,57 [18].

We observed, as well as all the studies in the literature, that inter and intra-observer reliability decrease if the classification is more complex, in fact kappa values strongly depends on the numbers of levels of classification investigated [18, 20, 24].

Our work, to our knowledge, is the first in literature considering the reliability of 2018 AO/OTA classification. A recent study analyzed this classification, but only for the extra capsular femur fractures (31A): simplified AO k value was 0,479, complete AO k value was 0,376 [24].

Masionis et all describe a k value for intraobserver reliability from 0,26 to 0,48 and a k value from 0,11 to 0,43 for interobserver reliability of the previous AO classification [18]; Blundell et al. found AO system had fair agreement [25]; Gaspar et al. calculated a k value of 0,17 for interobserver reliability [21].

Thus, it is important to notice that radiographic images were graded using the latest version of AO classification (2018); despite its complexity, it has a reliability higher than the previous version. Another strength of our study is that the reliability was analyzed considering the experience of observers. In literature, this particular analysis has been described only for Garden classification and for the previous version of AO classification [12, 18, 20].

Our results are similar to data founded in literature for the reliability when comparing more experienced to less experience surgeons [18, 20]. Data we founded favor opinion that experience does not improve the interobserver and intra observer reliability. This can be due to the learning curve of classifying fracture that is steeper in the first couple of years of practice and then decreases [12]; trauma residents making part of this study had already 3 years of experience in treatment this type of fractures.

Authors are aware of limitations of the present study. First, the low sample size of the evaluating surgeons. Then, it’s a retrospective study and patient outcomes were not evaluated. All the observers work in the same hospital and in the same university orthopedic department, it could have probably uniformed their classification. This is not a multicentric study, because patients were selected from a single department and consequently radiographic images were collected using the same protocol. Lastly, we considered only three classifications; we excluded other classifications, such as Pauwels and Waldenström i.e., because in the clinical practice these are the most common used.

## Conclusion

The latest version of AO classification (2018), despite its complexity, has a reliability higher than the previous version. Furthermore our results are similar to data founded in literature for the reliability when comparing more experienced to less experience surgeons. Garden and AO/OTA simplified classification are more reliable than AO/OTA classification with subgroups, in fact also in previous literature, inter and intra-observer reliability decrease when the classification become more complex. It does not mean that these classifications can be considered successful because their inter observer reliabilities are not high enough and even trauma experience did not improve them.

## Data Availability

The datasets used and/or analysed during the current study are available from the corresponding author on reasonable request.

## Abbreviations

FNF: Femoral neck fractures
AO: Arbeitsgemeinschaft für Osteosynthesefragen
OTA: Orthopaedics Trauma Association
CRIF: closed reduction internal fixation
ORIF: open reduction internal fixation
AO/OTA: AO Foundation/Orthopaedic Trauma Association
